# The Role of Resolvin D1 in the Differential Diagnosis of Pancreatic Ductal Adenocarcinoma and Acute Pancreatitis: A Case-Control Study

**DOI:** 10.3390/medicina61020168

**Published:** 2025-01-21

**Authors:** Yasemin Pekmezci, Sefa Ergun, Basar Can Turgut, Seyma Dumur, Ugurcan Sayili, Hafize Uzun, Salih Pekmezci, Mehmet Velidedeoglu

**Affiliations:** 1 Department of General Surgery, Cerrahpaşa Faculty of Medicine, Istanbul University-Cerrahpasa, Istanbul 34098, Turkey; dryaseminpekmezci@gmail.com (Y.P.); sefaergn@yahoo.com (S.E.); basarcanturgut@gmail.com (B.C.T.); pekmezcisalih@gmail.com (S.P.); m-veli@hotmail.com (M.V.); 2 Department of Medical Biochemistry, Faculty of Medicine, İstanbul Atlas University, Istanbul 34403, Turkey; seyma_dumur@hotmail.com; 3 Department of Public Health, Cerrahpasa Faculty of Medicine, Istanbul University-Cerrahpasa, Istanbul 34098, Turkey; ugurcan.sayili@iuc.edu.tr

**Keywords:** pancreatic cancer, metastatic pancreatic cancer, acute pancreatitis, marker, resolvin D1

## Abstract

*Background and Objectives*: Pancreatic ductal adenocarcinoma (PDAC) is an aggressive malignancy characterized by a dense desmoplastic stroma with a poor prognosis. The aim of this study was to investigate whether resolvin (Rv) D1 could be used as a potential serum biomarker to discriminate between PDAC and acute pancreatitis (AP). *Materials and Methods*: In total, 67 patients were enrolled in the present study, including 21 patients with resectable PDAC, 23 patients with metastatic PDAC, 23 patients with AP, and a control group of 21 healthy individuals. RvD1 levels of PDAC patients were also analyzed through ELISA at the 6th postoperative month. *Results*: The mean RvD1 was 1169.24 ± 285.99 in the control group, 885.04 ± 134.25 in the AP group, 728.57 ± 140.1 in the PDAC group, and 670.09 ± 105.6 in the metastatic pancreatic cancer (PC) group. RvD1 was significantly lower in PDAC and metastatic PC groups compared to controls and patients with AP, while it was significantly lower in patients with AP compared to the control groups. Postoperative RvD1 levels of patients with PDAC were significantly higher than preoperative levels (728.57 ± 140.1 vs. 885.43 ± 275.57). In the ROC analysis, when the cut-off value for serum RvD1 level was 825 ng/L, it was found to predict PDAC from metastatic PC with 84.1% sensitivity and 81.8% specificity. *Conclusions*: Serum RvD1 is a new biomarker for the detection of PDAC. Serum RvD1 may provide an important diagnostic contribution in clinical practice to predict PDAC. Serum RvD1 levels were found to be predictive with high sensitivity and specificity in differentiating PDAC from metastatic PC. However, it was concluded that serum RvD1 levels cannot be used as a detection marker to differentiate PDAC from AP. RvD1 could be a representative agent of a new class of drugs to be proposed for innovative treatment of AP and PDAC. Our future study will investigate whether RvD1 can be a marker to differentiate from chronic pancreatitis.

## 1. Introduction

Pancreatic cancer (PC) currently ranks fourth among cancer-related deaths in developed countries and is projected to rise to second place by 2030. There are 10 major types of neoplasms in PC. Pancreatic ductal adenocarcinoma (PDAC) accounts for approximately 85% of all PCs and originates from ductal epithelial cells. Metastasis is the primary cause of death in PDAC cases [1,2]. Despite advances in medicine and increased screening programs, PC is a group of cancers that is difficult to diagnose early and has very low 5-year survival [3]. At presentation, 52% of patients are diagnosed with distant metastasis and 23% with locally advanced disease [4].

Pancreatitis is a clinical picture characterized by inflammation in the pancreas that can lead to pathological events of varying severity, ranging from mild disease requiring conservative treatment to acute pancreatitis (AP), which may be associated with local and systemic complications [5]. The two most common causes of AP are cholelithiasis (35–40%) and alcohol (30%), but autoimmune pancreatitis, hypertriglyceridemia, endoscopic retrograde cholangiopancreatography (ERCP), some genetic mutations, drugs, and pancreatic duct injury are among the causes [6].

Resolvin D1 (RvD1), a derivative of docosahexaenoic acid (DHA), an omega-3 fatty acid, is an endogenous chemical mediator that exerts a potent anti-inflammatory effect and mediates the resolution of inflammation. RvD has been shown to limit neutrophil infiltration, stimulate phagocytosis and increase clearance through its receptors on macrophages, decrease the expression of cell surface proteins, and stimulate the M2 phenotype of monocytes/macrophages and microglial cells [6,7]. In colon cancer, RvD1 decreases as inflammatory cytokines and the cancer stage increase [8].

For a disease as aggressive and late-diagnosed as PC, early diagnosis becomes even more important. Although prognostic factors related to the disease have been defined, new markers are needed. For this reason, recent studies have reported prognostic markers defined at the molecular level for PC [9,10]. Resolvins are also expected to be important mediators of carcinogenesis due to their important role in the successful resolution of inflammation, as uncontrolled chronic inflammation is known to be associated with the development of solid malignancies [11,12,13,14]. However, there are limited data on the importance of immune resolvents in the development of PDAC. Few clinical studies have investigated this bioactive lipid (resolvin D) in patients with pancreatic adenocarcinoma, and the results are controversial [15].

In this study, we investigated the diagnostic value of serum resolvin D1 levels, a bioactive lipid, in PDAC.

## 2. Material and Methods

All subjects who participated in the study gave informed consent, and a case-control study was approved by the ethics committee of Istanbul University-Cerrahpasa, Cerrahpas Medical Faculty (approval number: 842919; Date: 22 November 2023). This study was conducted in accordance with the Declaration of Helsinki.

The sample size was calculated using G*Power 3.1.9.7. The parameters were set as follows: alpha error probability (α) = 0.05, power (1 − β) = 0.8, number of groups = 4, and a high effect size (>0.40). A high effect size was chosen due to the lack of prior studies in the literature on this specific topic. Based on these parameters, the minimum required sample size was determined to be 76 participants, with 19 participants per group. To account for potential data loss or other unforeseen circumstances, the target sample size was increased to at least 21 participants per group.

Our study consists of patients admitted to the hepatopancreatobiliary (HPB) clinic of Istanbul University-Cerrahpaşa, Cerrahpaşa Medical Faculty, General Surgery. The study included patients who were diagnosed with resectable PC (n = 21) and metastatic PDAC (n = 23) because of the evaluation and patients who were operated on in the general surgery clinic with the diagnosis of resectable PC (pathology result of PDAC) and who came to the 6th-month follow-up. The control group consisted of patients who were examined in our clinic and diagnosed with AP.

Healthy volunteers were another control group, who were age- and gender-matched to the patients. Volunteers who came to the hospital for a check-up without any disease were included in the health control group.

### 2.1. Inclusion Criteria

The inclusion criteria are as follows: (i) >18 years old; (ii) patients with diagnosed resectable PC (with a prediagnosis of PDAC); (iii) diagnosed patients with metastatic PDAC; (iv) patients who came to the 6th-month follow-up after PC surgery; and (v) patients and healthy volunteers who accepted and signed the informed consent form

### 2.2. Exclusion Criteria

The exclusion criteria are as follows: (i) <18 years old; (ii) patients whose pathology result after biopsy or surgery is not PDAC; (iii) patients and healthy volunteers who do not accept and sign the informed consent form; (iv) those with cancer and/or a history of cancer other than PDAC and metastatic PDAC; (v) among all groups, pregnant women and people with diabetes mellitus, a diagnosis of inflammatory bowel disease, acute/chronic infections other than AP, chronic pancreatitis, a diagnosis of inflammatory disease, obesity, and hyperlipidemia; (vi) people who have received chemotherapy or radiotherapy; and (vii) people taking omega-3 supplements.

All patients with PDAC preoperatively underwent a dedicated contrast-material-enhanced multi-detector-row computed tomography with a pancreatic protocol, as previously described [16].

Pathologic confirmation of PDAC diagnoses of the entire patient group was performed. The imaging and pathology reports of all patients were reviewed, and the location of the tumor in the pancreas, the size, the grade, and, in patients with metastatic PDAC, the location of metastasis were additionally recorded. Tissue biopsies of patients were performed by the same pathologist.

The AP group consisted of patients who presented to our clinic with abdominal pain. AP was diagnosed according to the Revised Atlanta Criteria (2012) [17]. Patients with more than 3-fold higher amylase and/or lipase levels, characteristic abdominal pain, and two-thirds of the imaging findings were considered to have AP and included in the study.

Venous blood samples were collected from the forearm of all participants between 08.00 and 10.00 h in the morning following fasting, centrifuged, and stored at −80 °C until the time of analysis. The levels of biochemical parameters in the separated serum samples were assayed on the same day using the autoanalyzer (Hitachi Modular System, Roche Diagnostic, Corporation, Hague Road, Indianapolis, IN, USA).

### 2.3. Measurement of Serum Resolvin D1 (RvD1) Levels

Serum RvD1 levels were assessed using a commercially available human-enzyme-linked immunosorbent assay (ELISA) kit (Human Resolvin D1 ELISA Kit, Bioassay Technology Laboratory, Katalog numarası: E7450Hu, Shanghai, China) according to the manufacturer’s instructions. All samples were examined twice. The sensitivity of the kit is 19.01 ng/L, and the measurement range is 37.5–2400 ng/L. Precision, intra-assay: CV < 8%; inter-assay: <10%.

Tumor markers were measured using IMMULITE 2000 (DPC, Los Angeles, CA, USA).

### 2.4. Statistical Analysis

Data analysis was conducted using SPSS software version 21.0 (IBM Corp., Armonk, NY, USA), and Jamovi v.2.4.11 was utilized for graphical representation. Categorical variables were expressed as frequencies (n) and percentages (%), while continuous variables were presented as mean ± standard deviation or median (25th–75th percentiles) based on distribution characteristics. The Shapiro–Wilk test, along with skewness and kurtosis values, were used to assess the normality of continuous data.

For comparisons of categorical variables, the chi-square or Fisher’s exact test were applied, as appropriate. Continuous variables across four independent groups were analyzed using one-way ANOVA for normally distributed data and the Kruskal–Wallis test for non-normally distributed data. Post hoc analyses involved Tukey’s test following significant ANOVA results, and adjusted *p*-values were reported for significant findings from the Kruskal–Wallis test. In comparisons between two independent groups, the independent samples t-test was used for normally distributed data, while the Mann–Whitney U test was applied for non-normal distributions. To compare preoperative and postoperative measures within the PC cohort (paired analysis), paired t-tests or Wilcoxon signed-rank tests were employed based on data normality.

Correlations between two continuous variables were evaluated through Pearson correlation analysis when both variables were normally distributed; otherwise, Spearman correlation was applied. Statistical significance was set at *p* < 0.05.

## 3. Results

Of the control group, 52.4% were female, as were 52.2% in the pancreatitis group, 52.4% in the PC group, and 47.8% in the metastatic PC group. Gender distribution among patient groups showed no significant difference (*p* = 0.987). The mean age was 58.48 ± 10.85 years in the control group, 59.65 ± 12.39 years in the pancreatitis group, 63.1 ± 9.6 years in the PC group, and 63.17 ± 9.42 years in the metastatic PC group, with no significant differences among these groups (*p* = 0.353).

Amylase levels differed significantly between patient groups. The mean amylase level was 68.67 ± 15.21 in the control group, 81.71 ± 62.12 in the PC group, 53.22 ± 23.78 in the metastatic PC group, and 1923.77 ± 964.14 in the pancreatitis group. Amylase levels in the pancreatitis group were significantly higher than those in the other groups. The median CEA and CA19-9 levels in the PC and metastatic PC groups were significantly higher compared to the control and pancreatitis groups. Significant differences in RvD1 levels were observed across all groups. The mean RvD1 level was 1169.24 ± 285.99 in the control group, 885.04 ± 134.25 in the pancreatitis group, 728.57 ± 140.1 in the PC group, and 670.09 ± 105.6 in the metastatic PC group. RvD1 levels were significantly lower in the pancreatic and metastatic PC groups than in the control and pancreatitis groups and significantly lower in the pancreatitis group compared to the control group (Table 1).

Among patients with PC, no significant difference was found between preoperative and postoperative amylase and CEA levels. Preoperative CA19-9 levels in PC patients were significantly higher than postoperative levels (330.25 [188–967.6] vs. 67 [45–152]). Preoperative serum RvD1 levels were significantly lower than postoperative levels (728.57 ± 140.1 vs. 885.43 ± 275.57) (Table 2).

The mean amylase levels of the control group and the postoperative PC group showed no significant difference. However, the postoperative CEA and CA19-9 levels in the PC group were significantly higher than those in the control group. Postoperative serum RvD1 levels in the PC group were significantly lower than those in the control group (Table 3).

Comparisons of biomarkers by cancer grade in PC patients showed no significant differences in any parameter between grade 2 and grade 3 (Table 4).

**Figure 1:**
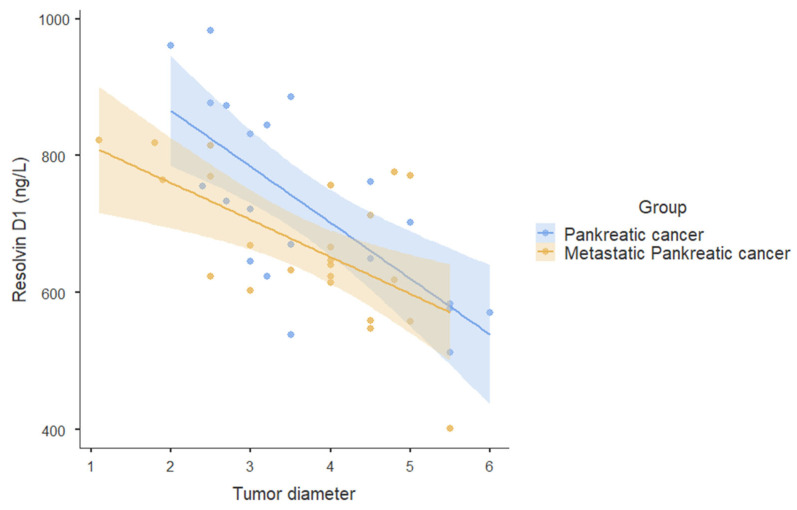
Correlation graph between tumor diameter and resolvin D1.

A strong negative correlation was found between tumor size and RvD1 levels in the PC group (r = −0.718; *p* < 0.001). No significant correlations were observed between tumor size and age, amylase, CEA, and CA19-9 levels or between RvD1 and age, amylase, CEA, and CA19-9 levels (Table 5, Figure 1).

In the metastatic PC group, a moderate negative correlation was observed between tumor size and RvD1 levels (r = −0.513; *p* = 0.012). No significant correlations were found between tumor size and age, amylase, CEA, and CA19-9 levels or between RvD1 and age, amylase, CEA, and CA19-9 levels (Table 5, Figure 1).

**Figure 2:**
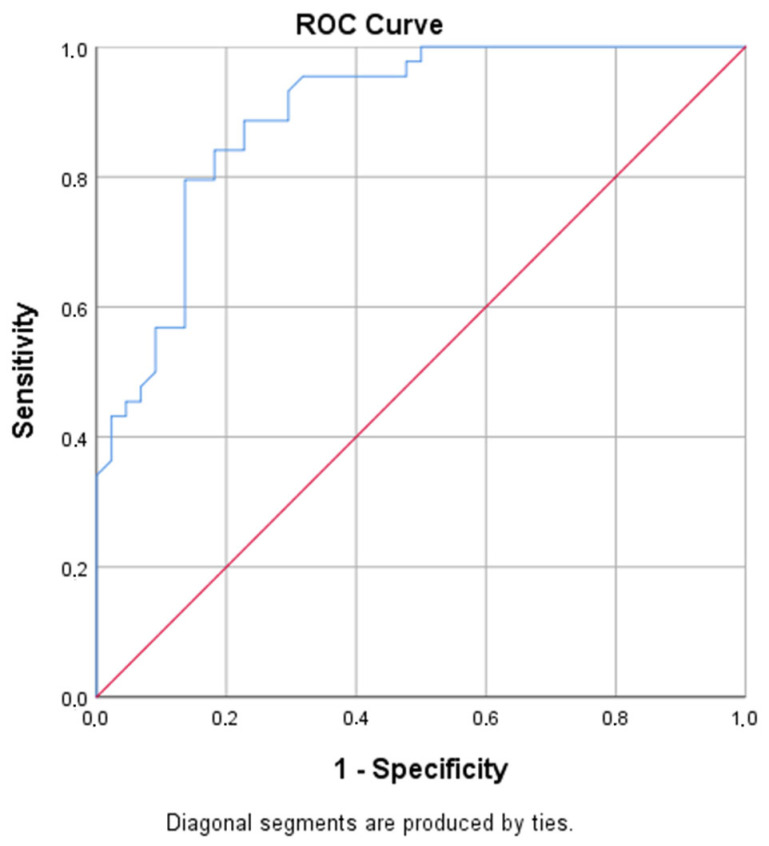
The ROC curve of resolvin D1 and pancreatic cancer.

The ROC analysis of RvD1 levels for pancreatic/metastatic PC yielded an area under the curve (AUC) of 0.895, indicating significance as a parameter. A RvD1 level below 825 ng/L had 84.1% sensitivity and 81.8% specificity for pancreatic/metastatic PC; a level below 900 ng/L had 95.5% sensitivity and 61.4% specificity; and a level below 1000 ng/L achieved 100% sensitivity with 38.6% specificity (Table 6, Figure 2).

## 4. Discussion

In the present study, the diagnostic value of serum RvD1 levels, a bioactive lipid, in PDAC was investigated. We found that patients with PDAC had significantly reduced serum RvD1 levels. The lowest RvD1 level among the study groups was found in the metastatic group. The highest value was found in the healthy group, and it was significantly lower in the pancreatitis group compared to the healthy group, as in the other groups. These results may lead to an increased risk of unresolved inflammation, tumor formation, and tumor cell invasiveness due to inadequate production of anti-inflammatory lipid mediators (systemic RVD1).

Resolvins (RVs) have potent anti-inflammatory and anti-carcinogenic properties. Blogowski et al. [15] compared systemic concentrations of lipoxins (A4 and B4) and resolvins (D1 and D2) in patients with pancreatic adenocarcinoma (n = 68) with healthy subjects. They also investigated the relationship between clinical TNM staging of PDAC and immunoresolvent levels in patients. They reported that immunoresolvents, such as lipoxins and resolvins, were elevated in patients with PDAC, which is different from our results. They emphasize that there are significant changes in the systemic balance of lipoxins and resolvins and that this finding is not affected by the clinical TNM stage of the disease. They also suggested that their study is the first to measure peripheral levels of lipoxin and resolvins (especially RvD1) in humans, which may be novel biomarkers of PDAC. In the present study, serum RvD1 levels were higher in the PDAC group than in the metastatic PDAC group. Our results showed that systemic RvD1 levels progressively decreased as inflammation progressed and the cancer became metastatic. These results suggest that unresolved inflammation due to inadequate production of anti-inflammatory lipid mediators (systemic RvD1) may lead to an increased risk of tumor formation and cell invasiveness.

RvD1, an endogenous anti-inflammatory lipid mediator, has recently been found to exert anti-cancer effects by acting on stroma cells [18]. RvD1 impaired paracrine of cancer-associated fibroblast (CAF)-derived cartilage oligomeric matrix protein (COMP) by targeting formyl peptide receptor 2 (FPR2)/reactive oxygen species (ROS)/forkhead box M1 (FOXM1) signaling to repress epithelial–mesenchymal transition (EMT) and cancer stemness in hepatocellular carcinoma (HCC). Thus, it has been reported that RvD1 may be a promising therapeutic option in HCC [18]. In the present study, serum RvD1 levels were significantly lower in PDAC and metastatic PDAC groups compared to healthy patients and patients with AP. As a novel anti-inflammatory mediator targeting tumor stroma, RvD1 may be a useful agent in combination with conventional therapy to improve treatment outcomes in PDAC patients. A strong negative correlation was also found between tumor diameter and RvD1 levels in the PC group, while a moderate negative correlation was found between tumor diameter and RvD1 levels in the metastatic PC group. Therefore, RvD1 has great potential as an alternative noninvasive method for early PC detection.

Among PC biomarkers, carbohydrate antigen 19-9 (CA 19-9) is the most extensively evaluated [19]. According to a study by Kim et al. [20], among 1063 patients with elevated serum CA 19-9 levels in 70,940 asymptomatic individuals, only 4 PC patients were identified. Although the sensitivity and specificity were 100% and 98.5%, respectively, it gave a poor positive predictive value (PPV) of only 0.9%. Satake et al. [21] found elevated serum CA 19-9 levels in 8706 of 12,840 asymptomatic patients with symptoms suspicious for PC, including weight loss, epigastric pain, and jaundice. Among 18 asymptomatic patients (0.2%) with elevated CA 19-9 levels, only 4 PCs (1 resectable) were detected. Among 8706 patients, 198 patients (4.3%) had elevated serum CA 19-9 levels. After comprehensive examination, PC was detected in 85 patients (1.8%), of whom 28 (0.4%) were resectable [22]. In the present study, the CEA and CA19-9 levels of the PDAC and metastatic PDAC groups were significantly higher than those of the healthy and pancreatitis patients. Serum CA 19-9 is the most comprehensively verified PK biomarker, with numerous clinical applications. The serum CA19-9 levels have a sensitivity and specificity of 79–81% and 82–90%, respectively, for the diagnosis of PC in symptomatic patients. However, it is not useful as a screening marker due to its low PPV (0.5–0.9%) [23]. However, 10–15% of patients with low or normal CA 19-9 levels preoperatively may have unresectable disease on examination; similarly, 5–10% of patients with high CA 19-9 levels preoperatively will be resectable [23,24]. Halloran et al. [25] found an unresectable disease that was considered resectable according to radiologic criteria in 17 (21%) of 80 patients with low serum CA 19-9 levels (<37 U/mL). While preoperative serum CA 19-9 provides good prognostic information about the PC stage, it should not be the sole criterion for determining resectability to avoid false negative or false positive surgical exploration [23,26,27].

AP is a potentially fatal disease with high morbidity and mortality rates, characterized by wide clinical variation from a mild, self-limiting disease to severe disease complicated by sepsis and multiorgan failure [28,29]. Today, despite the development of new diagnostic tools and treatment options, several problems remain in the management of severe AP. AP is a type of specialized inflammatory disease that can lead to systemic inflammatory responses (SIRS) that cause “more severe damage in distant organs than in the pancreas”. This is typically observed in the clinic [30,31]. Patients with chronic pancreatitis have been recognized to be at high risk of developing PC. This therefore provides solid evidence for a link between pancreatic inflammation and PC [32]. The persistent inflammation dysplasia–intraepithelial neoplasia–pancreatic adenocarcinoma sequence has been proven and is associated with loss of tumor suppressor genes and mutation of proto-oncogenes [33]. Wang et al. [34] reported in an experimental study that RvD1 ameliorated the severity of cerulein-induced acute pancreatitis in mice due to reducing impaired autophagy and restoring autophagic flux. Intramuscular diclofenac reduces the incidence of pancreatitis after endoscopic retrograde cholangiopancreatography (ERCP). It has been reported that this may be related to the ability of diclofenac to increase RvD1 and RvE1 levels [35]. In the present study, RvD1 was found to be significantly lower in PDAC and metastatic PDAC groups compared to healthy and AP patients but lower in AP patients compared to healthy groups. The present results suggest that RvD1 may be used as a therapeutic target in AP and distant organ damage. Further studies on the functions of RvD1 in inflammatory pathways and mechanisms associated with AP may facilitate the search for novel therapeutic targets for the treatment of AP and related distant organ damage.

Studies have shown that the history of AP may accelerate carcinogenesis in PDAC [36,37]. Thus, the relationship between pancreatitis and PC development has been clearly demonstrated. Therefore, early detection of patients with suspected cancer during pancreatitis is of great importance. Atypical PDAC features include findings related to associated conditions, such as acute or chronic pancreatitis, an isointense mass in the parenchyma, density, diffuse tumor infiltration, associated calcifications, and cystic components. Various neoplastic and inflammatory conditions can mimic PDAC, including paraduodenal “groove” pancreatitis, autoimmune pancreatitis, focal acute and chronic pancreatitis, neuroendocrine tumors, solid pseudopapillary neoplasms, metastases, and lymphoma. Differentiating these conditions from PDAC can be difficult due to the overlap of CT and MRI features; however, some findings may help differentiate them. Diffusion-weight MRI may be helpful but nonspecific [38]. In a study conducted in Turkey in 2020 by Gül-Utku et al. [39], they reported that serum RvD1 and CA 19-9 levels can be used to effectively differentiate between benign biliary diseases (BBDs) and cholangiocarcinoma (CCA); both RvD1 and CA 19-9 levels are associated with the stage of CCA, and they can also be used to assess disease progression. According to the ROC analysis performed in present study, when the cut-off value for the serum RvD1 level was 825 ng/L, it was found to predict PDAC from metastatic PDAC with 84.1% sensitivity and 81.8% specificity. However, it was shown that it cannot be used to differentiate AP from PDAC. These results need to be confirmed by further studies in which the number of patients with both cancer and pancreatitis is increased.

In addition to suggesting a therapeutic role for RvD1 based on experimental studies, the use of synthetically produced resolvins as therapeutics to treat respiratory diseases has shown promising preclinical results [40,41,42]. However, pro-resolving therapies are still in their infancy, and some limitations must be resolved for successful clinical application. Most notably, resolvins are considered unstable, and they are quickly metabolized and degraded. This limits the bioavailability of the drug and has led to impracticable scenarios, such as repetitive administration to assess therapeutic effects during experimentation [43,44].

### Limitations of the Study

Because the role of RvD1 with anti-inflammatory properties was investigated in our study, careful selection of the patient group and exclusion of patients with acute infections or chronic diseases other than AP to eliminate possible confounders of inflammatory processes are the strengths of our study. However, the small number of patients included in the study is a limitation. In light of all of these data, large population studies in which RvD1 and inflammatory cytokines can be investigated are needed to determine the role of RvD1 in the etiopathogenesis of PC. In addition, the inability to perform multivariate analyses due to multicollinearity should be considered as a limitation.

## 5. Conclusions

Compared with healthy volunteers, the levels of serum RvD1 in AP, PDAC, and metastatic PDAC patients decreased significantly. Serum RvD1 may provide an important diagnostic contribution in clinical practice to predict PDAC. A negative correlation was found between tumor diameter and RvD1 levels in the PDAC group, while a negative correlation was found between tumor diameter and RvD1 levels in the metastatic PDAC group. Therefore, serum RvD1 seems to have potential as an alternative noninvasive marker for early PC detection and evaluation of recurrences. RvD1 may be used as a non-invasive biomarker that can be used to distinguish between patients with PDAC and metastatic PDAC. However, it was concluded that serum RvD1 levels cannot be used as a detection marker to differentiate PDAC from AP. In the PC group, preoperative serum RvD1 levels significantly decreased compared to postoperative levels. Therefore, RvD1 may be useful in postoperative clinical follow-up to detect early recurrence. Decreased RvD1 levels play a role in carcinogenesis by affecting tumor formation and inflammatory pathways. Therefore, systemic RvD1 may be a contributing factor in reducing inflammation in PDAC and AP patients. RvD1 may therefore be a representative agent of a new class of drugs to be proposed for the innovative treatment of AP and PDAC. These results suggest that enhancing the endogenous clearance of tumor cell debris using RvD1 is a novel therapeutic target that could complement cytotoxic cancer therapies. In our future study, we plan to research whether RvD1 will be a marker to differentiate from chronic pancreatitis. Moreover, longitudinal studies and larger cohorts are needed to confirm these findings.

## Figures and Tables

**Table 1:** Comparison of biomarkers of patient groups.

		Control	Pancreatitis	Pancreatic Cancer	Metastatic Pancreatic Cancer	*p* Value
Amylase	Mean ± SD	68.67 ± 15.21 ^a^	1923.77 ± 964.14 ^b^	81.71 ± 62.12 ^a^	53.22 ± 23.78 ^a^	<0.001 †
	Median (25th–75th percentile)	70 (58–78)	1812 (1091.8–2647)	74 (40–96)	57 (38–65)	
CEA	Mean ± SD	2.17 ± 1.49	1.64 ± 0.97	27.91 ± 63.81	20.7 ± 27.36	<0.001 ●
	Median (25th–75th percentile)	1.69 (1.14–2.92) ^a^	1.33 (0.99–2.29) ^a^	5.47 (2.62–9.78) ^b^	7.74 (3.66–25.6) ^b^	
CA19-9	Mean ± SD	12.63 ± 11.75	24.02 ± 19.67	742.76 ± 910.09	16,641.52 ± 44,863.18	<0.001 ●
	Median (25th–75th percentile)	9.51 (5.65–15.48) ^a^	23.1 (6.37–34.1) ^a^	330.25 (188–967.6) ^b^	1355 (251–9314) ^b^	
AFP	Mean ± SD	3.3 ± 4.55	3.05 ± 2.03	3.3 ± 2.73	2.54 ± 1.29	0.808 ●
	Median (25th–75th percentile)	2.32 (1.54–2.9)	3.1 (1.37–4.5)	2.8 (1.83–3.24)	2.21 (1.66–3.53)	
Resolvin D1 (ng/L)	Mean ± SD	1169.24 ± 285.99 ^a^	885.04 ± 134.25 ^b^	728.57 ± 140.1 ^c^	670.09 ± 105.6 ^c^	<0.001 †
	Median (25th–75th percentile)	1142 (991–1357)	888 (817–978)	722 (623–845)	647 (614–769)	

†: One-way ANOVA Test; ●: Kruskal–Wallis test. Different superscript letters indicate significant differences.

**Table 2:** Comparison of preoperative and postoperative biomarkers in the pancreatic cancer group.

	Preoperative		Postoperative		
	Mean ± SD	Median (25th–75th Percentile)	Mean ± SD	Median (25th–75th Percentile)	*p* Value
Amylase	81.71 ± 62.12	74 (40–96)	57.57 ± 23.97	56 (48–68)	0.134 *
CEA	27.91 ± 63.81	5.47 (2.62–9.78)	47.01 ± 155.92	6.5 (3.58–9.4)	0.510 *
CA19–9	742.76 ± 910.09	330.25 (188–967.6)	674.11 ± 1717.13	67 (45–152)	0.019 *
AFP	3.3 ± 2.73	2.8 (1.83–3.24)	3.84 ± 1.03	3.71 (2.76–4.61)	0.027 *
Resolvin D1 (ng/L)	728.57 ± 140.1	722 (623–845)	885.43 ± 275.57	942 (682–1047)	0.023 †

†: Paired *t*-test; *: Wilcoxon test.

**Table 3:** Comparison of postoperative results of the pancreatic cancer group and the control group.

	Control		Pancreatic Cancer—Postoperative		
	Mean ± SD	Median (25th–75th Percentile)	Mean ± SD	Median (25th–75th Percentile)	*p* Value
Amylase	68.67 ± 15.21	70 (58–78)	57.57 ± 23.97	56 (48–68)	0.081 †
CEA	2.17 ± 1.49	1.69 (1.14–2.92)	47.01 ± 155.92	6.5 (3.58–9.4)	<0.001 *
CA19-9	12.63 ± 11.75	9.51 (5.65–15.48)	674.11 ± 1717.13	67 (45–152)	<0.001 *
AFP	3.3 ± 4.55	2.32 (1.54–2.9)	3.84 ± 1.03	3.71 (2.76–4.61)	0.001 *
Resolvin D1 (ng/L)	1169.24 ± 285.99	1142 (991–1357)	885.43 ± 275.57	942 (682–1047)	0.002 †

†: independent samples *t*-test; *: Mann–Whitney U test.

**Table 4:** Comparison of biomarkers according to grades.

	Grade 2 (n: 16; 76.2%)		Grade 3 (n: 5; 23.8%)		
	Mean ± SD	Median (25th–75th Percentile)	Mean ± SD	Median (25th–75th Percentile)	*p* Value
Amylase	88.31 ± 66.75	79.5 (42–99.5)	60.6 ± 43.05	59 (32–74)	0.364 *
CEA	35.05 ± 72.08	6.26 (3.66–20.47)	5.03 ± 4.19	2.62 (1.94–9.4)	0.342 *
CA19–9	776.49 ± 977.5	320.13 (170–1135.97)	634.82 ± 734.21	372 (330–516)	0.804 *
AFP	3.5 ± 3.06	2.81 (1.84–3.4)	2.66 ± 1.15	2.3 (1.83–2.93)	0.967 *
Resolvin D1 (ng/L)	752.81 ± 136.11	744 (648–861)	651 ± 137.14	623 (577–670)	0.137 *
Amylase (Postoperative)	54.69 ± 17.58	53.5 (46–65.5)	66.8 ± 39.69	65 (63–68)	0.322 *
CEA (Postoperative)	59.93 ± 177.94	6.78 (4.08–9.13)	5.65 ± 3.77	5.25 (2.62–9.4)	0.563 *
CA19-9 (Postoperative)	719.88 ± 1937.05	64 (37.85–140)	527.66 ± 798.03	113 (53.2–516)	0.620 *
AFP (Postoperative)	3.67 ± 1.05	3.61 (2.72–4.6)	4.41 ± 0.83	4.32 (3.81–4.61)	0.137 *
Resolvin D1 (Postoperative) (ng/L)	905.06 ± 287.88	971 (702.5–1047)	822.6 ± 249.88	711 (634–1047)	0.620 *

*: Mann–Whitney U test.

**Table 5:** Correlation of tumor diameter and resolvin D1 with age and other biomarkers.

			Tumor Diameter	Resolvin D1 (ng/L)
Pancreatic cancer	Resolvin D1 (ng/L)	r	−0.718	
		*p* value	<0.001	
	Age	r	−0.212	0.144
		*p* value	0.356	0.533
	Amylase	r	0.220	0.128
		*p* value	0.338	0.581
	CEA	r	0.026	0.187
		*p* value	0.911	0.417
	CA19-9	r	0.081	−0.064
		*p* value	0.728	0.782
	AFP	r	−0.147	0.009
		*p* value	0.524	0.969
Metastatic pancreatic cancer	Resolvin D1 (ng/L)	r	−0.513	
		*p* value	0.012	
	Age	r	0.120	0.048
		*p* value	0.586	0.828
	Amylase	r	−0.407	−0.072
		*p* value	0.054	0.745
	CEA	r	0.364	−0.150
		*p* value	0.087	0.494
	CA19-9	r	0.004	0.194
		*p* value	0.986	0.376
	AFP	r	0.024	0.089
		*p* value	0.915	0.685

The relationships between age, RVD1, and amylase were evaluated through Pearson correlation analysis, and other relationships were evaluated through Spearman correlation analysis.

**Table 6:** ROC analysis results for pancreatic and metastatic pancreatic cancer.

Variable	AUC	95% CI	*p* Value	Cut-Off	Sensitivity	Specificity
Resolvin D1	0.895	0.831–0.960	<0.001	825 *	84.1%	81.8%
				900 *	95.5%	61.4%
				1000 *	100%	38.6%

*: Less than value. Analyses were performed as pancreatic cancer/metastatic pancreatic cancer vs. healthy/pancreatitis groups.

## Data Availability

The data underlying this article are available in the article. If needed, please contact the corresponding author. The email address is huzun59@hotmail.com.
